# Tandem repeat polymorphisms are associated with brain structure: results of two large population-based studies

**DOI:** 10.1186/s13073-026-01702-1

**Published:** 2026-07-06

**Authors:** Richard Mantey, Jialu Hu, Maryam Touhidinia, Tanzeem Butt, Ahmed Mokhtar Sidky, Roohollah Sobhani, Santiago Estrada, Kristian Haendler, Elena De Domenico, Marc Daniel Beyer, Monique Maria Bernadette Breteler, Nasir Ahmad Aziz

**Affiliations:** 1 Population Health Sciences, German Center for Neurodegenerative Diseases (DZNE), Bonn, Germany; 2 School of Computer Science, Northwestern Polytechnical University, Xi’an, China; 3 AI in Medical Imaging, German Center for Neurodegenerative Diseases (DZNE), Bonn, Germany; 4 Platform for Single Cell Genomics and Epigenomics (PRECISE), German Center for Neurodegenerative Diseases (DZNE) and University of Bonn and West German Genome Center, Bonn, Germany; 5 Institute for Human Genetics, University Hospital Schleswig-Holstein, Lübeck, Germany; 6 Immunogenomics and Neurodegeneration, German Center for Neurodegenerative Diseases (DZNE), Bonn, Germany; 7 Institute for Medical Biometry, Informatics and Epidemiology (IMBIE), University of Bonn, University Hospital Bonn, Bonn, Germany; 8 Centre of Neurology, Clinic for Parkinson, Sleep and Movement Disorders, University of Bonn, University Hospital Bonn, Bonn, Germany

**Keywords:** Genome-wide association studies, Short tandem repeats, Missing heritability, Brain imaging-derived phenotypes, Rhineland Study, UK Biobank

## Abstract

**Background:**

Although genome-wide association studies (GWAS) have uncovered many genetic variants linked to brain structure, much of its heritability still remains unexplained. Short tandem repeats (STRs) are rarely considered in GWAS but may account for part of this “missing heritability”. While the causal association of large pathogenic repeat expansions with a range of brain disorders is well established, the role of non-pathogenic STR variations in the general population is largely unknown. In this study, we systematically assessed the relationship between STR variations and brain imaging-derived phenotypes across the adult lifespan in the general population.

**Methods:**

We used targeted deep sequencing to genotype approximately 3,000 polymorphic STRs across 2,958 individuals (mean age: 54.1 years, range: 30–90 years, 57.1% women) from the population-based Rhineland Study in Bonn, Germany. STR sizes at 2940 loci were estimated using ExpansionHunter v5, while 45 brain imaging-derived phenotypes were obtained from 3T T1-weighted MRI scans using the FreeSurfer processing pipeline. Associations between STR lengths and neuroimaging phenotypes were assessed using multiple linear regression models, adjusting for age, sex, population stratification, and other relevant covariates. Significant findings were independently assessed for directional consistency in the UK Biobank Imaging Substudy (*N* = 38,879), leveraging available whole-genome sequencing data.

**Results:**

The expansion of an intronic AC repeat in *PRR14L* was associated with larger thalamic volume (standardized β [95% CI] = 0.15 [0.06–0.24]), while AATG repeat polymorphisms in *NADK* were associated with reduced subcortical gray matter volume (–0.05 [–0.08 to − 0.01]) and thalamic volume (–0.06 [–0.08 to − 0.04]). These associations were directionally consistent in the UK Biobank cohort. Beyond single loci, higher polygenic burden of moderate STR expansions was associated with increased total brain, gray matter, supratentorial, and thalamic volumes (all multiple-testing–corrected *p* < 0.05).

**Conclusions:**

Our findings indicate that moderate STR expansions are region-specific determinants of brain morphology and suggest that STR variability may have evolved to enhance neuroanatomical plasticity and cognitive function. By leveraging large population-based cohorts, our study extends current understanding of how repetitive genomic elements contribute to inter-individual variation in brain structure beyond the effects of single-nucleotide variation.

**Supplementary Information:**

The online version contains supplementary material available at 10.1186/s13073-026-01702-1.

## Background

A large proportion of the risk for common age-associated brain disorders, such as Alzheimer’s and Parkinson’s disease, is genetically determined. Over the past few decades, genome-wide association studies (GWAS) have provided insights into the genetic underpinnings of many neurodegenerative and age-related brain diseases [1–4]. However, a substantial amount of the genetic basis of these diseases still remains unknown. A potential cause of this “missing heritability” conundrum is that many GWAS have predominantly focused on the role of single nucleotide polymorphisms (SNPs), often neglecting the potentially important contributions of other sources of genomic variation. Repetitive regions of the human genome such as retrotransposable elements and short tandem repeats (STRs) are among the most polymorphic structural genetic variants and may account for part of the “missing heritability” problem of brain disorders.

STRs, also referred to as microsatellites, are generally defined as DNA sequences consisting of repeated motifs of 1–6 base pairs [5]. These conspicuous sequences are not only ubiquitous in the human genome but are also substantially more polymorphic than SNPs due to replication slippages [6]. Variations in STRs copy numbers therefore contribute substantially to genetic diversity [7]. STRs are particularly known for their genomic instability, which often leads to dynamic changes in repeat length across generations as well as within somatic tissues [8]. Indeed, genomic instability-induced repeat expansion in certain genes is thought to have given rise to repeat expansion disorders (REDs), most of which are characterized by neurological abnormalities [8]. Even though the pathological consequences of large repeat expansions are well documented, much less is known about the potential effects of repeat length variation within the normal or premutation range of the respective loci. This knowledge gap is particularly striking for brain-related phenotypes in the general population. STRs are prone to somatic expansion over time due to replication-associated mutations, which suggests that even subtle variations in repeat length could influence brain structure and function across the lifespan [9]. Understanding these dynamics could therefore provide valuable insights into the broader role of STR variations as determinants of brain structure, particularly in the context of aging.

An important implication of age-related somatic instability is the possibility that STR variants exert age-dependent effects, particularly on cognition and other brain-related traits. We previously demonstrated that cytosine-adenine-guanine (CAG) repeat length variations in three polyglutamine disease-associated genes (PDAGs) were significantly associated with cognitive decline [10]. Furthermore, we observed that large normal-range CAG repeats in *TBP* and *ATXN7* genes were linked to an increased lifetime risk of depression [11], while those in *AR* and *ATXN1* gene modified disease expression in Alzheimer’s disease [12]. Collectively, these findings underscore the potential role of STRs in modulating brain function even below the pathogenic expansion threshold associated with REDs.

The direction and magnitude of the effects of non- and sub-pathological allele lengths in REDs genes, however, remain to be fully elucidated. Interestingly, the Kids-HD study, a longitudinal study of early manifestations of Huntington’s disease (HD) in children and young adults, demonstrated that CAG repeat mutation carriers in the *HTT* gene exhibited significantly better cognitive, behavioral, and motor performance than non-expanded gene carriers in early life. The characteristic structural and functional brain decline emerged later, following this initial peak in brain performance [13]. These results align with the evolutionary theory of antagonistic pleiotropy, which was first proposed by Williams in 1957 and has been refined and supported by recent findings from other species [14]. This theory posits that certain genetic variants that may confer advantages early in life could become detrimental in later years [15]. This may be particularly the case for some human-specific STR loci that initially may have evolved to confer advantages in cognitive capacity [16–18].

The examples detailed above represent only a minute fraction of the vast STR landscape. The human genome contains hundreds of thousands of STRs whose potential influence on brain-related traits remains largely unexplored. Therefore, here we aimed to systematically investigate the relationship between STR polymorphisms and brain imaging-derived phenotypes across the adult lifespan in two large, well-characterized, population-based cohorts of European ancestry. Our findings indicate that moderate STR expansions, particularly in genes linked to neurodegeneration, can indeed act as region-specific determinants of brain morphology and provide further support for the antagonistic pleiotropy hypothesis of STR expansions in humans.

## Methods

### Experimental design

This population-based research was conducted using two well-characterized cohorts to investigate the contribution of STR variation to individual differences in brain structure. The study followed a two-stage design: discovery in the Rhineland Study cohort (*N* = 2,958) and replication in the UKB cohort (*N* = 38,879). With this setup, we aimed to identify specific STR loci and polygenic STR patterns associated with brain imaging-derived phenotypes.

### Cohorts description

#### Rhineland study

We used data from the first 2,958 participants of the Rhineland Study – an ongoing population-based cohort study in Bonn, Germany – in whom both DNA samples and brain imaging-data were available. The Rhineland Study recruits individuals from two geographically defined districts in Bonn and aims to investigate the etiology, risk factors and predictive biomarkers of aging and age-associated (brain) diseases [19]. Eligible participants aged 30 years and older are invited to the study center, where they undergo comprehensive physical examinations and in-depth (endo)phenotyping. To ensure clear understanding of the study procedures and informed consent, sufficient proficiency in German is required for inclusion. The protocol of Rhineland Study was approved by the ethics committee of the University of Bonn Medical Faculty (Ref: 338/15). The study is conducted according to the International Conference on Harmonization Good Clinical Practice standards (ICH-GCP), with written informed consent obtained in accordance with the Declaration of Helsinki.

#### UK biobank study

The UKB is one of the largest population-based cohort studies to date, including over 500,000 participants. The study was launched in 2006 with invitations sent to 9.2 million eligible individuals aged 40–69 years living in England, Scotland, and Wales. Approximately 500,000 participants enrolled by attending one of 22 assessment centers across the UK, where baseline data were collected through touchscreen questionnaires, verbal interviews, and physical assessments. Biological samples were also obtained to enable genotyping and a wide range of hematological and biochemical assays [20, 21]. In 2014, the UKB launched its imaging initiative, inviting participants back for multimodal imaging assessments as part of a follow-up visit [22]. For the present study, we included 49,304 participants who completed their first imaging visit, of whom 45,306 also had WGS data available. We chose the UKB to assess the directional consistency of STR–brain imaging-derived phenotype associations in an independent population with large-scale, harmonized imaging and genomic data. While differences in sequencing and genotyping approaches may influence absolute STR lengths, the analysis was intended to evaluate consistency in the direction and pattern of observed effects. Approval for conducting UKB Study was received from the National Information Governance Board for Health and Social Care and the National Health Service North West Centre for Research Ethics Committee (Ref: 11/NW/0382). Every participant provided written informed consent. This research was conducted under project ID 82056.

### Targeted STR panel design

For participants of the Rhineland Study with available DNA samples obtained from whole blood, an amplicon-based targeted sequencing approach was applied for the genotyping of 2940 TR-containing regions. These 2,940 polymorphic STR loci were selected through a multi-step curation process. First, we compiled STRs previously implicated in repeat expansion disorders using publicly available databases (i.e., STRipy, ExpansionHunter catalog, and disease-specific literature) [23, 24]. Second, we identified STRs located within or proximal (± 10 kb) to genes associated with neurodevelopment or brain structure based on Gene Ontology annotations and prior comparative genomics studies [17, 18, 25–33]. Finally, we incorporated highly polymorphic forensic STR markers (i.e., CODIS and Marshfield panels) to ensure representation of genome-wide variability. A complete list of all selected STR loci, including genomic coordinates, gene annotations, and selection category, is provided in Additional file 3. Subsequently, a custom targeted amplicon panel targeting these loci was designed in collaboration with Illumina Concierge Design Services using DesignStudio to maximize in-silico coverage and optimize primer configurations. All loci and assay regions were selected to ensure comprehensive coverage of the intended targets.

### DNA extraction and library preparation

Genomic DNA was extracted from peripheral blood samples of 2958 participants of the Rhineland Study using standard magnetic bead-based protocols and quantified through Qubit fluorometry [34]. Libraries were prepared using the AmpliSeq Library PLUS kit (Illumina, 384 reactions per kit) following the manufacturer’s recommendations [35]. For each reaction, 50–100 ng of genomic DNA was used as input. Multiplex PCR was performed for targeted amplification of the panel regions. Following amplification, PCR products underwent enzymatic primer digestion and magnetic bead-based purification. Purified amplicons were indexed using the AmpliSeq CD Index Set A-D, enabling assignment of unique dual indices to 96 samples per set to facilitate multiplexing. Indexed libraries were quantified (Qubit) and normalized for pooling. Equal molar pools were prepared for sequencing following Illumina’s guidelines. We measured the size-distribution using the Agilent high sensitivity D5000 assay on a TapeStation 4200 system (Agilent technologies).

### Targeted sequencing

Pooled libraries were sequenced on an Illumina NovaSeq 6000 platform using the SP Reagent Kit (500-cycle configuration, 2 × 250 bp paired-end reads). Given the amplicon-based design, the average insert size was approximately 120 bp, corresponding to the targeted amplicon length. Sequencing achieved a targeted mean coverage depth of 180×. While ~ 30× coverage is typical for whole-genome sequencing, higher depth is advantageous for targeted STR sequencing and was therefore prioritized in this study. All sequencing procedures were performed according to the manufacturer’s workflow for denaturation, dilution, and loading (Illumina, NovaSeq 6000 System Guide). Quality control was performed on the FASTQ files using FastQC with default parameters (Version 0.11.9), after which the high quality reads were aligned to the human reference genome (GRCh38) using the BWA-mem algorithm (version 0.7.17) [36, 37]. The diploid genotypes in all 2940 loci were then estimated with ExpansionHunter version 5 using our custom variant catalog [38].

### UK Biobank whole genome sequencing protocol

This study utilized available WGS data from the UKB, which was performed on the Illumina NovaSeq 6000 platform using 2 × 150 bp paired-end reads, with a mean coverage of approximately 32.5× per sample and standard fragmentation-based library preparation. Sequence reads were mapped to the GRCh38 human reference genome using the BWA algorithm. The study design, sample selection, and bioinformatic pipeline have been extensively detailed previously [39, 40]. To perform STR genotyping in the UKB cohort, we implemented ExpansionHunter version 5 as a custom applet and deployed it through the DNAnexus toolkit on the UKB Research Analysis Platform. This setup enabled efficient and scalable genotyping of STRs directly from the aligned WGS data.

### Custom variant catalog design for targeted STR genotyping

The ExpansionHunter algorithm requires a variant catalog, which is a structured JSON file that specifies the genomic coordinates, locus structure, and repeat motif of the loci to be genotyped. Publicly available catalogs exist for genome-wide STR genotyping and for disease-specific pathogenic loci. However, to reduce computational demands and restrict analyses to loci of interest, we generated a custom variant catalog. To build this catalog, we first identified genomic intervals containing exact repeats of DNA motifs (1–10 bp in length) across the autosomes of the GRCh38 reference genome. This was achieved using our in-house tool STRfinder/STRbean (v1.0), applied to the selected STR targets [41, 42]. To filter out low quality STR loci, we implemented a filtering step in STRfinder using the sequence complexity of flanking regions. Sequence complexity was estimated using Shannon entropy and sequence repetitiveness, and loci with low diversity were excluded from downstream analyses [43]. Each identified locus was then annotated based on GENCODE v38 (https://www.gencodegenes.org/human/release_38.html). The STRfinder output, generated in BED format, was subsequently converted into a JSON-formatted variant catalog compatible with ExpansionHunter using customized scripts [44]. The final catalog contained 2,817 polymorphic STR loci (excluding sex chromosomes), which were then used for downstream genotyping. Notably, this customized catalog was consistent with those produced by recently published STR catalog generation protocols [44]. Additional file 1: Fig. S1 shows the distribution of STR tract lengths across the genomic regions included in our customized panel, as annotated in the human reference genome.

### Quality-control of genotyped STRs

The approach utilized in the STR genotyping and filtering steps followed established methodologies [23, 45, 46]. Briefly, we run ExpansionHunter version 5 separately on each individual BAM file with default parameters using our custom variant catalog (as described above). The individual ExpansionHunter VCF outputs were subsequently sorted, compressed and indexed using bcftools (version 1.15.1) [47]. The indexed files were further processed using the DumpSTR algorithm from the TRTool Suite (6.1.0) to perform call-level and locus-level filtering as published [46, 48]. Additionally, the parameter --eh-min-call-LC 10 was included in DumpSTR algorithm to remove low quality calls. Taken together, these steps effectively removed low quality STRs as well as loci whose genotypes did not follow Hardy-Weinberg Equilibrium (at *p* < 0.001). Moreover, as an additional quality-control step, we applied REViewer (version 0.2.7) to a subset of randomly selected ExpansionHunter output files to visualize read alignments at individual loci [49]. This step was particularly important for evaluating genotype calls where the repeat region exceeded the read length or where the ExpansionHunter-genotyped confidence intervals were wide, enabling a more accurate assessment of genotyping quality. The same STR genotyping and quality-control pipelines were applied to both cohorts. For the UKB cohort, the variant catalog included only loci corresponding to the top STR association hits for each phenotype, forming a subset of the main variant catalog.

### Outlier and rare expansion detection

STR genotyping benefits from longer sequencing read lengths to accurately resolve repeat tracts. In the Rhineland Study, sequencing reads were approximately three times longer than those in the UKB. However, challenges in accurately calling repeat lengths persisted particularly for expansions approaching or exceeding the read length. To enhance the reliability of STR calls, we employed a density-based spatial clustering of applications with noise (DBSCAN) approach, as described previously [50]. DBSCAN is a non-parametric clustering algorithm that identifies dense clusters of data points - here, repeat length estimates - while flagging isolated points in low-density regions as potential outliers. This method enabled robust identification of individuals harboring unusually long STR alleles for each locus, thereby minimizing the inclusion of spurious or artefactual repeat calls.

### Polygenic STR burden

To assess polygenic STR burden, we performed a stratified analysis using predefined tract length thresholds of ≥ 1, ≥5, ≥ 10, and ≥ 20 repeat units longer than the GRCh38 reference genome. We considered the longer of the 2 alleles at each locus. The longer allele was used in that context because the burden framework was designed to capture the cumulative effect of expanded alleles exceeding biologically motivated thresholds for known repeat expansion disease loci and graded increase in expansion magnitude for the other loci with unknown pathogenic threshold. At each threshold, we quantified the number of STR loci exceeding the specified repeat length for each individual which we referred to as polygenic STR expansion burden metric. This score was then tested for association with brain imaging-derived phenotypes while controlling for covariates stated in the Statistical Analyses section. This approach follows a prior study of STR expansion burden [50]. Because the pathogenic tract length is unknown for the vast majority (~ 95%) of STR loci included in our panel, biologically validated, locus-specific expansion thresholds are currently unavailable. Hence, we used this method to capture a gradient of expansion severity.

### Imaging data

In the Rhineland Study, we used a bespoke imaging protocol to acquire MRI images from participants at two study sites. This involved the use of two identical 3T MRI scanners, each equipped with 64-channel head-neck coils (MAGNETOM Prisma; Siemens Healthcare). The detailed imaging protocol of the Rhineland Study has been described previously [51]. In this study, we utilized only the 0.8 mm isotropic T1-weighted (T1w) scans from the Rhineland Study-acquired sequences. The Rhineland Study T1w protocol employs a multi-echo magnetization prepared rapid gradient echo (MPRAGE) sequence [52] with 2D acceleration [53]. Detailed sequence parameters are provided in Additional file 1: Table S1.

Participants from the UKB imaging sub-study were invited to one of three dedicated imaging centres, each equipped with an identical 3T Siemens Skyra scanner [54]. The MRI acquisition protocol was harmonized across sites, with identical hardware configuration and standardized examination procedures. The UKB imaging protocol has been described previously [54, 55], and all acquired sequences are summarized in Additional file 1: Table S2. Consistent with the Rhineland Study dataset selection, only T1w scans were analysed in the current study [55]. UKB 1 mm isotropic T1w images were acquired using a 3D MPRAGE sequence with the following parameters: repetition time (TR) = 2000 ms, echo time (TE) = 2.01 ms, inversion time (TI) = 880 ms, flip angle = 8°, and field of view (FOV) = 208 × 256 × 256 mm³ [55].

For both cohorts, T1w images were then processed using the FreeSurfer pipeline (v6.0), a neuroimaging software package for automated cortical and subcortical segmentation, to derive imaging-derived phenotypes for subsequent analyses [56]. A total of 45 brain imaging-derived phenotypes, primarily consisting of volumetric and subcortical brain measures, were tested in our genetic association analysis. Although the acquisition protocols differed between the two cohorts, the T1w sequences used follow state-of-the-art acquisition standards, such as MPRAGE, which is highly recommended for morphometric studies because of its superior gray/white matter contrast [52]. Moreover, differences in native image resolution between cohorts are unlikely to substantially affect downstream analyses, as the standard FreeSurfer processing pipeline resamples all scans to a uniform 1 mm isotropic resolution.

### Gene ontology enrichment analysis

GO enrichment analysis was conducted in the R statistical environment (v4.1.0). Prior to this, we performed a genome-wide annotation of approximately 1.2 million STRs identified in the GRCh38 human reference genome. To determine the genomic context of each STR, we performed a proximity search against annotated human genes from the UCSC Genome Browser (GRCh38 reference genome) using the TxDb.Hsapiens.UCSC.hg38.knownGene package. The goal was to identify the nearest gene for each STR locus. Only STRs located within a maximum distance of 250 kb from a gene’s TSS were retained for further analysis. This approach ensured that the analysis focused on potentially regulatory or functionally relevant loci. Finally, the Entrez IDs of these nearest genes were subsequently mapped to their official gene symbols using the org.Hs.eg.db annotation package [57]. This method ensured that all STRs were associated with their closest gene and that the gene names were standardized for downstream interpretation. Next, we evaluated the genes in our custom STR panel at predefined expansion thresholds based on carrier frequency within each cohort. At each threshold (≥ 1, ≥ 5, ≥10, or ≥ 20 repeat units longer than the reference), we identified the most frequent genes shared between cohorts and used these for gene set enrichment analysis. Subsequently, functional enrichment analysis was performed using the selected genes at the respective thresholds as input using gprofiler2 (v0.2.3) [58]. A custom background gene set, comprising all genes associated with the original STR reference panel, was used to ensure enrichment tests were limited to the relevant genomic space. This over-representation analysis was conducted against multiple databases, including GO Biological Process, GO Molecular Function, GO Cellular Component, the Kyoto Encyclopedia of Genes and Genomes (KEGG) and Reactome. Enrichment of GO and pathway terms was assessed using a cumulative hypergeometric test, and statistical significance was defined as *p* < 0.05 after correction for multiple testing using the FDR method. We note that the STR set analyzed in this study was selected using a hypothesis-driven approach, enriched for loci previously implicated in STR-associated disorders and genes involved in neurodevelopmental processes. Therefore, enrichment analyses were performed conditionally within this predefined STR panel rather than across the whole genome. Functional enrichment was assessed only among genes containing STRs meeting the predefined expansion thresholds within this targeted set.

### Statistical analyses

Demographic characteristics were summarized as means with standard deviations, while sex was reported as percentages. For the single association testing we used allele dosages, which was calculated as the mean of the two STR alleles genotyped at each locus. This is consistent with prior STR association studies as this provides a statistically stable representation for quantitative traits and avoids over-parameterization, particularly for loci with a wide range of allele lengths [59, 60]. Since most of the STR genotypes were not normally distributed, we first applied rank-based inverse normal transformation to these genotypes. We then performed multiple linear regression to assess the association between STR dosage and brain imaging-derived phenotypes, using STR dosage as a continuous variable (main independent variable) and the brain imaging-derived phenotypes as dependent variables. In each model, age was included as a covariate to account for well-established age-related variation and decline in global and regional brain volumes [61]. Sex was included to adjust for known sex differences in brain morphology and volumetric measures [62]. Extracted total intracranial volume (eTIV) was included in volumetric models to control for inter-individual differences in head size. To account for potential confounding due to population stratification, genetic principal components derived from genome-wide microarray data in the respective cohorts were included in the model. These components capture ancestry-related genetic variation and reduce the risk of spurious associations. To assess potential non-linearity in age effects on brain–imaging phenotypes, we compared linear and quadratic age models using likelihood ratio tests. In addition, we generated LOESS plots of covariate-adjusted outcomes (residuals after regressing out sex, intracranial volume, and genetic principal components) against age to visually evaluate deviations from linearity. Based on these analyses, the majority of phenotypes demonstrated non-linear age effects (37/45 in the Rhineland Study and 41/45 in the UKB). Accordingly, a quadratic age term was included in all models to flexibly capture potential non-linear age-related variation where present. Furthermore, sensitivity analyses were performed to evaluate the potential impact of imaging acquisition site. Inclusion of imaging site as a covariate in the Rhineland Study cohort did not materially alter the observed associations, with consistent effect directions and relative effect sizes retained. In the UKB cohort, imaging data were acquired using harmonized protocols and centralized quality control procedures; however, imaging site contributions were substantially imbalanced (Bristol: *n* = 54; Cheadle: *n* = 22,888; Newcastle: *n* = 10,530; Reading: *n* = 5,407). Given the robustness of the findings, together with the substantial site imbalance in UKB, imaging site was not included in the primary analytical models for either cohort. We report the estimates and p-values of the rank-based inverse normal transformed STR genotypes. To correct for multiple comparisons across the tested loci, we applied Bonferroni correction by setting the significance threshold to *p* < 0.05/2477 for the number of independent loci tested. For the polygenic STR burden, a total of 4 STR burden thresholds were evaluated across 45 imaging-derived phenotypes (180 nominal tests). The Li & Ji method was applied to estimate the effective number of independent imaging tests [63]. This method accounts for the correlation among the brain imaging-derived phenotypes. Based on the number of effective tests, the statistical significance threshold was set at 0.05/26 for the Rhineland Study and 0.05/22 for the UKB. The analysis in the UK Biobank aimed to assess directional consistency across loci identified in the discovery cohort. Directional consistency was defined by concordant effect directions between datasets and evaluated independently of statistical significance. STR loci showing concordant effects were considered directionally consistent, while those additionally meeting the Bonferroni-corrected threshold (α = 0.05/n) were considered statistically significant.

As a sensitivity analysis, we additionally performed 10,000 permutations of the STR burden predictors to empirically evaluate the adequacy and robustness of the multiple-testing correction under the correlated testing structure. Empirical family-wise error rate thresholds derived from the permutation analyses were broadly consistent with those obtained using the Li & Ji approach; therefore, the Li & Ji correction was retained as the primary multiple-testing framework.

## Results

### STR profiling in two independent cohorts

For discovery we utilized data from the first 2,958 participants of the population-based Rhineland Study in whom both brain imaging data and DNA samples were available (see Table 1 for participant characteristics). We performed targeted sequencing (with paired-end 2 × 150 bp reads) of ~ 3,000 STR loci using an amplicon-based targeted sequencing approach on the Illumina platform. For replication of our findings, we used the UKB as an independent cohort. Specifically, we focused on participants with available brain imaging data from the UKB imaging sub-study [22]. Of the ~ 50,000 individuals with imaging data available, whole genome sequencing (WGS) data were available for 45,306 participants. WGS in UKB was also conducted using the Illumina platform with ~ 30× coverage and 150-bp paired-end reads [40]. Unlike the Rhineland Study, which is a relatively homogeneous cohort of predominantly European ancestry, the UKB is more genetically diverse. Therefore, to minimize population structure differences between the cohorts, we restricted our replication analyses to 38,879 UKB participants of genetically determined British ancestry (Table 1).

**Table 1:** Participant characteristics in the Rhineland Study and UK Biobank Imaging Substudy

Variable	RS Cohort	UKB Cohort
Sample size (n)	2958	38,879
Age, mean (SD)	54.1 (13.5)	55.1 (7.5)
Age range (years)	30–90	40–70
Female, n (%)	1688 (57.1%)	20,518 (52.8%)

Participant characteristics in the Rhineland Study and UK Biobank Imaging Substudy

*Abbreviations*: *RS* Rhineland Study, *SD* standard deviation, *UKB* UK Biobank

### Population characteristics

Among participants included in the Rhineland Study, the mean age was 54.1 ± 13.5 years, with women comprising 57.1% of the sample. On the other hand, participants from the UKB study had a mean age of 55.1 ± 7.5 years, with 52.8% women. Additional characteristics of the brain imaging-derived phenotypes such as global measures (e.g., total brain volume, total gray matter volume) and subcortical structures (e.g., thalamus and related regions) are provided in Additional file 1: Table S3.

### Selection and characterization of STR loci for association testing

**Fig. 1:**
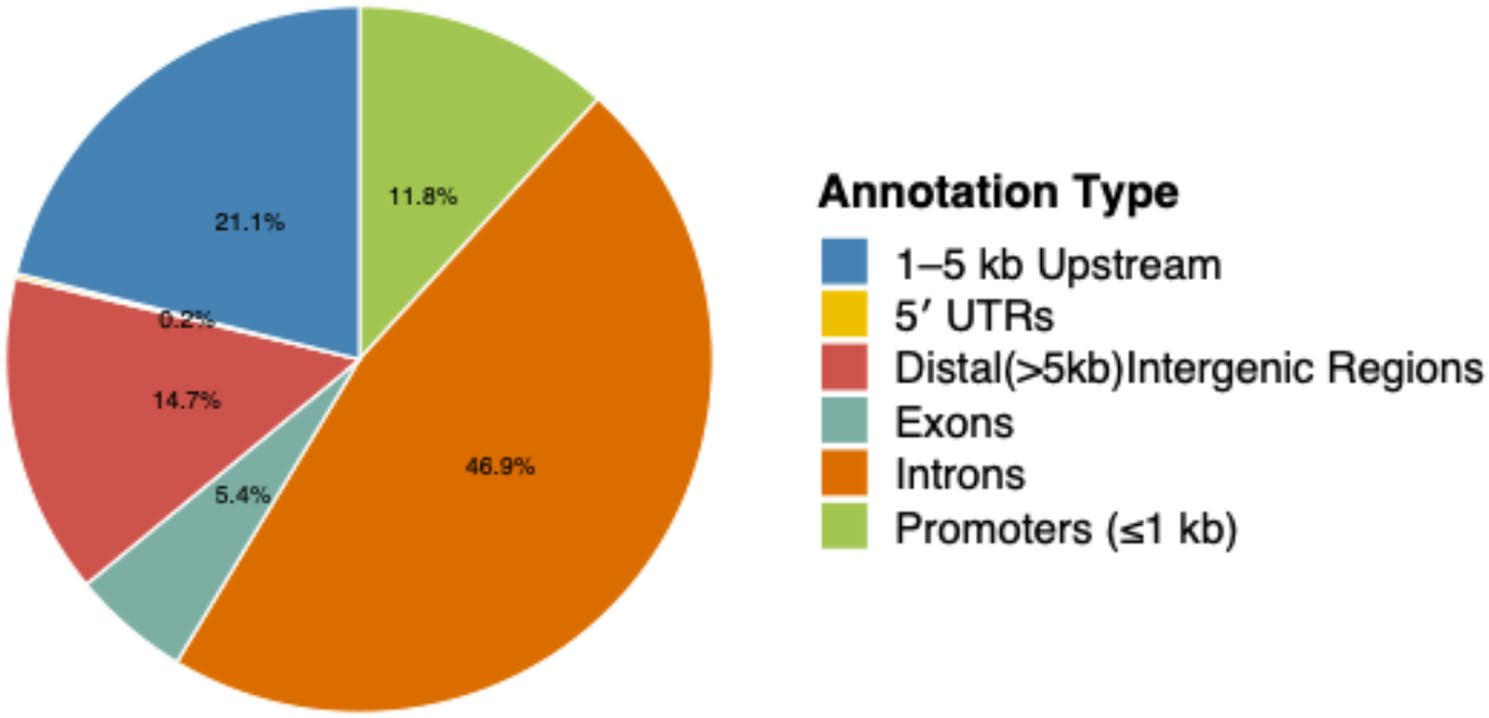
Genomic distribution of the 2,477 STR loci included in the customized STR panel analyzed in this study. STRs are classified by genomic location: intronic, intergenic, promoter, upstream (1–5 kb), exonic, and UTR. Abbreviations: UTR, untranslated region

To investigate the effect of STR polymorphisms on brain health, we first conducted comprehensive targeted genetic association analyses. Our initial customized STR catalogue included 2,817 loci which were carefully curated based on their previously reported roles in brain structure and function, as well as their potential association with neurological disorders as identified through comparative genomic studies [17, 18, 25–28]. Additionally, highly polymorphic STR loci commonly used in DNA fingerprinting and linkage studies – such as those included in the Marshfield genetic map – were included in our catalogue [64]. From this, we focused on 2,447 STRs that were polymorphic, defined here as showing variability in tract length across the participants of the Rhineland Study. The genomic distribution of these analysed STRs is summarized in Fig. 1. The majority of STRs (46.9%) were located within intronic regions, followed by distal intergenic regions (21.1%), defined as sequences outside gene bodies and ≥ 3 kb from the nearest transcription start site (TSS). Additionally, 11.8% were positioned 1–5 kb upstream of the TSS, while 14.7% resided within promoter regions (< 1 kb upstream of the TSS). Only a small proportion were located within exonic regions (5.4%) or untranslated regions (UTRs, 0.2%).

### Locus-specific genetic association analyses

**Fig. 2:**
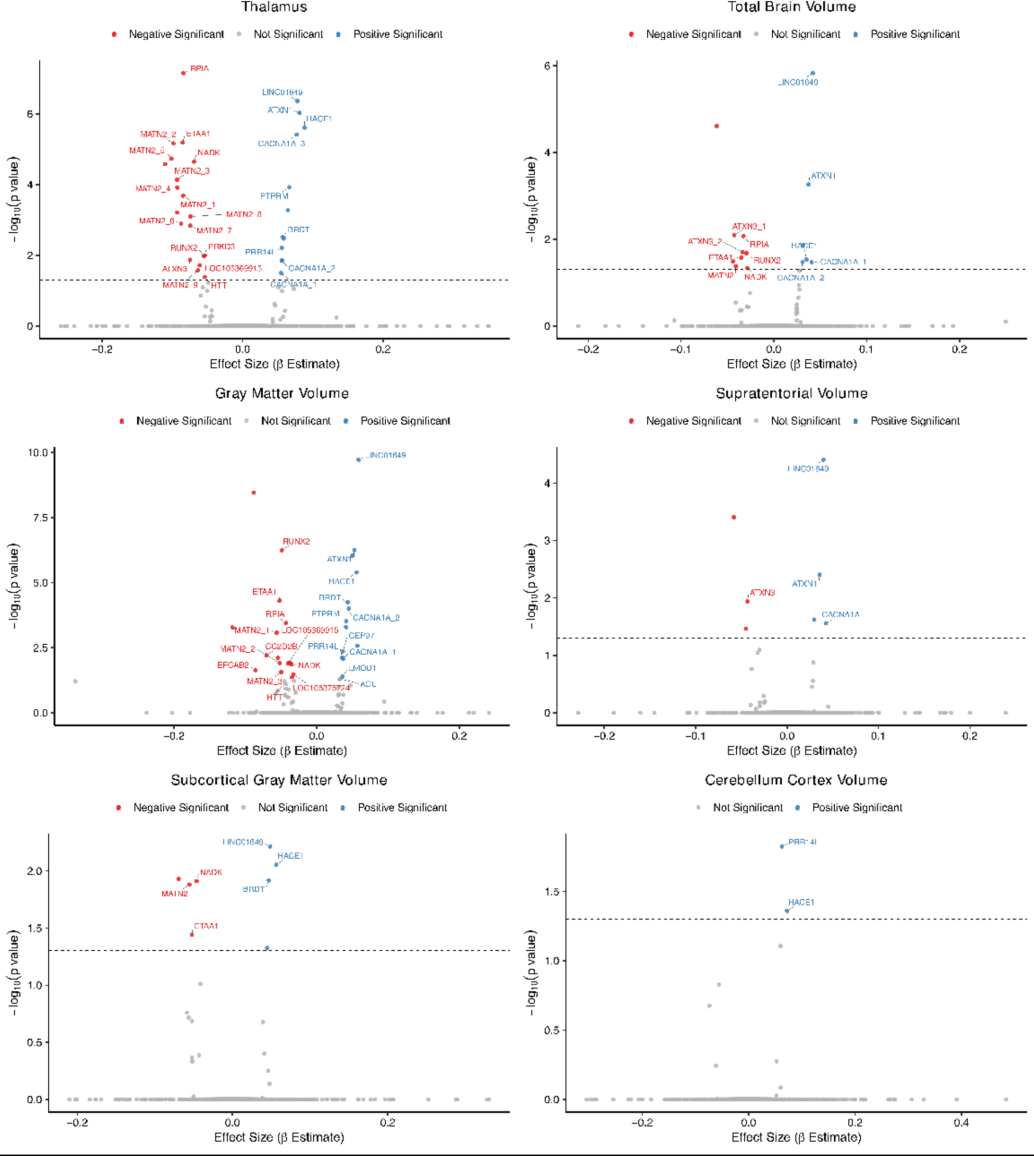
Associations between STR loci and regional brain volumes in the Rhineland Study cohort. Volcano plots depict the associations between STR loci and regional brain volumes. The x-axis represents the effect size (β estimates) and the y-axis the –log10 p-values. Black dashed lines indicate the FDR significance thresholds. Genes annotated in blue are positively associated with the respective brain imaging-derived phenotype, whereas those in red are negatively associated. Gene suffix numbers indicate distinct loci within the same gene to which short tandem repeats were mapped in the gene proximity analysis. Significant loci without gene annotations were not labeled for visual clarity. The corresponding genomic coordinates are provided in Additional file 2

In our genetic association analyses, we used allelic dosage, defined here as the mean repeat length of the two alleles per STR locus, as the primary independent variable in a multiple linear regression framework. We then applied a rank-based inverse-normal transformation to account for the non-normal distribution observed at most STR loci. All models were adjusted for age, sex, the first 10 genetic principal components as well as other relevant covariates as described in the ‘Statistical Analyses’ section of the Methods. In the Rhineland Study discovery cohort, we observed heterogeneous patterns of association between STR loci and brain morphology across multiple regions. While some loci were related to global volumetric measurements, others exhibited a more region-specific effect. Thalamic volume showed the strongest enrichment, with 31 significant associations, followed by total gray matter volume (30 loci) and total brain volume (15 loci) (Fig. 2). Additional associations were detected for supratentorial (7 loci) and subcortical gray matter volumes (8 loci), with several loci overlapping across brain regions (Fig. 2). Interestingly, some of the overlapping genes include both well-established disease-causing loci and novel candidates. For instance, extreme repeat expansions in some of these overlapping genes such as *ATXN1* and *CACNA1A* are known to cause spinocerebellar ataxia (SCA) types 1 (OMIM #164400) and 6 (OMIM #183086), respectively, in humans, whereas repeat length polymorphisms in others, including *PRR14L*, *RUNX2*, and *MATN2*, have not been previously implicated in neurological disorders. These findings highlight both known disease-associated STRs and novel candidates that may play broader roles in influencing brain structure.

**Fig. 3:**
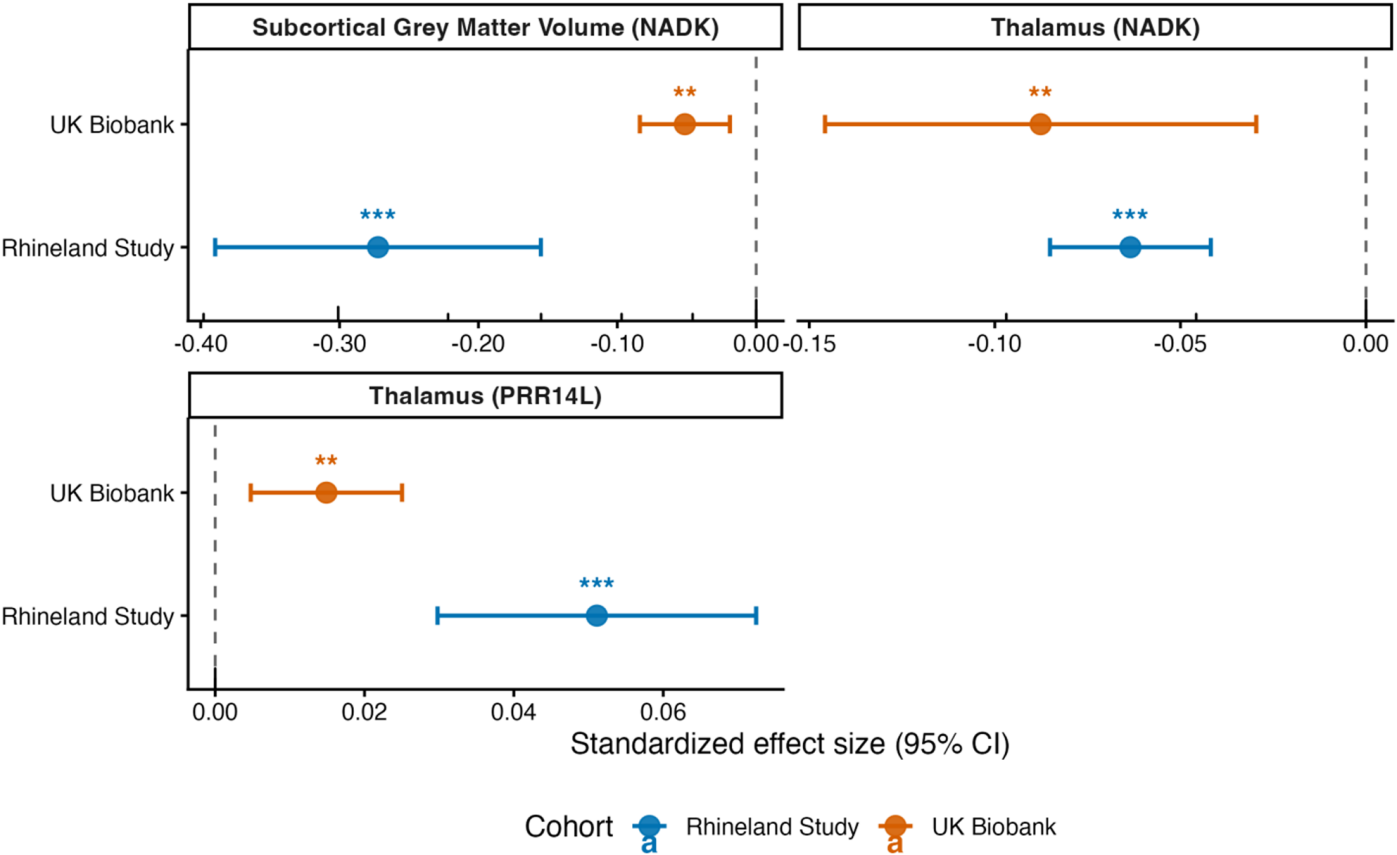
Bivariate confidence interval plot: Rhineland Study vs. UK Biobank. Plot shows standardized effect sizes for associations of an AATG repeat size in the *NADK* gene with subcortical grey matter volume and thalamus volume (upper panel), and an AC repeat size in the *PRR14L* gene with thalamus volme (lower panel), in the Rhineland Study (*n* = 2,958) and the UK Biobank Imaging Substudy (*n* = 38,879). Dots represent point estimates with 95% confidence intervals. ***p < 0.00001, **p < 0.005

To externally validate the identified associations, we tested the STRs that retained statistical significance after false discovery rate (FDR) correction in the Rhineland Study for association testing with the respective brain imaging-derived phenotypes in the UKB cohort. We applied a Bonferroni-adjusted threshold (α = 0.05/n, where n is the number of loci carried forward for follow-up analyses). STR loci in the UKB with concordant effect directions and p-values below this threshold were considered directionally consistent with the discovery findings. Across brain imaging-derived phenotypes, 63.5% of tested STR loci showed concordant effect directions between the Rhineland Study and the UKB cohort, of which 32% reached nominal significance (*p* < 0.05) (Additional file 1: Fig. S2). Furthermore, we identified consistent robust association signals for both thalamic and subcortical gray matter volumes. Specifically, AC repeat motif variations at chr22, located in the intronic region of the *PRR14L* gene, were significantly associated with larger thalamic volume (β = 0.15, 95% CI [0.06 to 0.24]) (Fig. 3). In addition, polymorphisms of an AATG repeat motif in *NADK* were significantly associated with reduced subcortical gray matter volume (β = − 0.05, 95% CI [–0.08 to − 0.01]) and thalamic volume (–0.06[–0.08 to − 0.04]). To facilitate direct comparison of effect sizes across cohorts, we constructed a bivariate confidence interval plot for the two significant STR thresholds (Fig. 3). The plot displays standardized effect estimates and corresponding 95% confidence intervals for thalamic volume and total subcortical grey matter volume in the Rhineland Study, alongside the corresponding estimates in the UKB. This visualization allows assessment of effect size magnitude, directionality, and cross-cohort concordance. The direction of association was consistent across cohorts for both traits, providing support for cross-cohort consistency of these associations, which may highlight their importance in brain morphology. A comprehensive list of all tested loci is provided in Additional file 1: Fig. S2.

The allele distributions for the *NADK* and *PRR14L* genes in the respective cohorts are presented in Additional file 1: Fig. S3. Although the overall distributions are comparable between cohorts, *PRR14L* shows a broader range of allele sizes in Rhineland Study compared with UKB. This observation may reflect population-specific differences in repeat length variation or technical differences in sequencing read length.

We further assessed whether the observed STR associations could be explained by linkage disequilibrium with nearby common variants. To this end, we examined SNPs within ± 250 kb of the lead STR loci and performed association analyses. No significant SNP associations were identified in these regions, suggesting that the STR signals are unlikely to be explained by nearby common SNPs captured in our data.

### Aggregated STR length and brain morphology

To assess the association of STR lengths aggregated over all 2477 tested loci with brain structure, we z-standardized the mean allele size at each locus and summed these values to derive an aggregate standardized mean allele score. This approach enabled capturing the cumulative variation in STR length across the target loci while reducing locus-specific scaling effects. The aggregated STR length was then included as the independent variable in the regression models with brain imaging-derived phenotypes as the dependent variables.

We observed that a higher aggregated STR length was associated with increased overall brain volume. While the association with total brain volume did not reach statistical significance in the Rhineland Study, it was significant in the UKB. Conversely, a larger aggregated STR length was significantly associated with increased thalamic volume in the Rhineland Study but did not reach statistical significance in the UKB (Additional file 1: Fig. S4). Importantly, however, the highly consistent direction and magnitude of effects across multiple brain regions and across both cohorts support a potential biological role of STR length variations in shaping brain morphology.

### Polygenic STR burden and brain morphology

In our primary genetic association analysis, we modeled STR tract lengths as continuous variables in relation to the brain imaging-derived phenotypes. We used this approach in order to capture the full spectrum of naturally occurring STR variation and its potential contribution to inter-individual differences in brain structure. Moreover, an intriguing aspect of allele length polymorphisms is understanding the influence of allele sizes that have not yet reached pathological thresholds on brain phenotypes. While pathogenic STR expansions are typically defined by well-established length thresholds associated with disease onset and severity, it remains unclear whether non-expanded or moderately expanded alleles contribute to the normal variation in brain morphology. Among the 2,817 STR loci included in our customized catalogue, fewer than 100 have known pathogenic thresholds. Therefore, rather than applying arbitrary cutoffs, we developed a STR polygenic burden as previously described by Guo and colleagues [50]. In this framework, we used pre-specified expansion thresholds relative to the GRCh38 reference genome (≥ 1, ≥ 5, ≥10, or ≥ 20 repeat units) for each locus. While these thresholds are not inherently pathogenic, they allowed us to evaluate whether associations were primarily driven by common alleles or by more extreme expansions in the general population. Unlike aggregated STR burden, which is calculated as the sum of standardized allele sizes across all target loci that captures the full spectrum of repeat-length variation across both shorter and longer alleles, polygenic STR burden reflects the cumulative number of only those loci that exceed a predefined expansion threshold, thereby specifically capturing the accumulated effects of expanded repeats within a subset of STR loci.

The distribution of polygenic STR expansion burden across the two cohorts, as well as the corresponding sex-stratified distributions, are shown in Additional file 1: Figs. S5, S6 and S7.

**Fig. 4:**
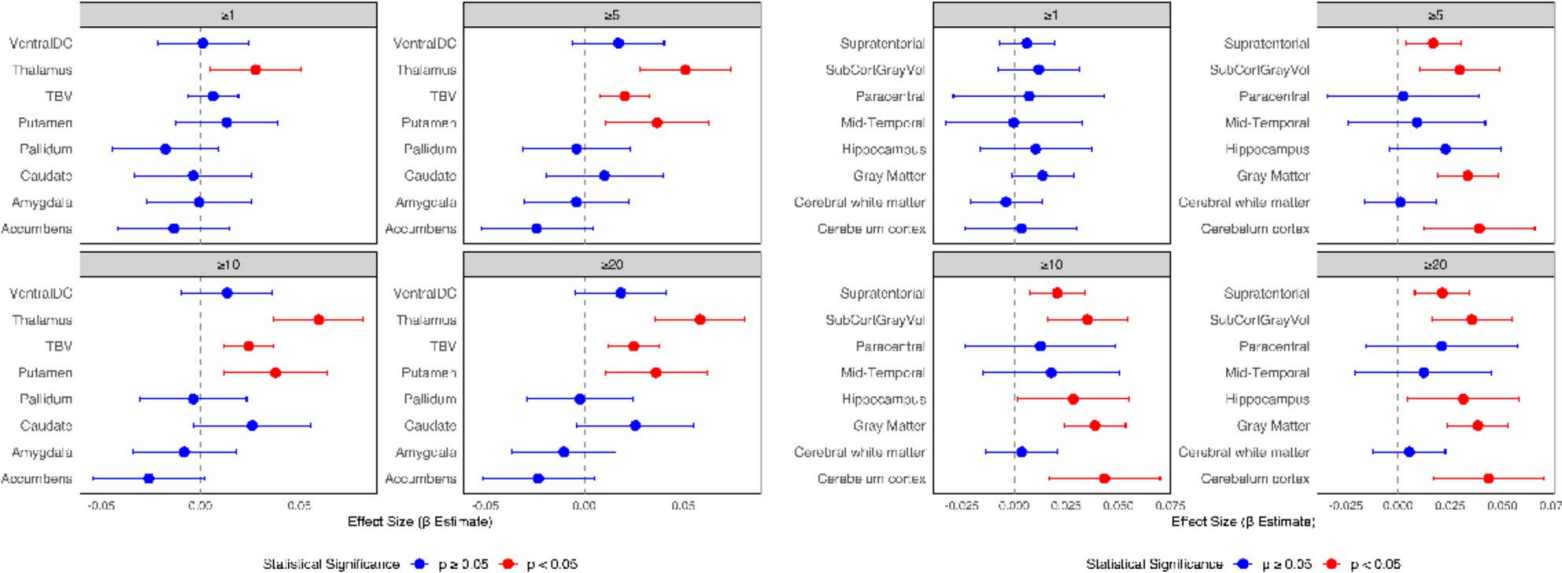
Polygenic STR expansion burden and brain phenotypes in the Rhineland Study. Forest plot of standardized regression coefficients (β) with 95% confidence intervals for the association between polygenic STR expansion burden and brain phenotypes in the Rhineland Study discovery cohort. Each point corresponds to the standardized β estimate for a given brain region at a specific STR threshold and the horizontal bars indicate 95% confidence intervals. Associations remaining after correction for multiple testing using the Li & Ji method are shown in red and non-significant associations are shown in blue. The vertical dashed line represents the null effect (β = 0). Abbreviations: TBV, total brain volume; ventral DC, ventral diencephalon

**Fig. 5:**
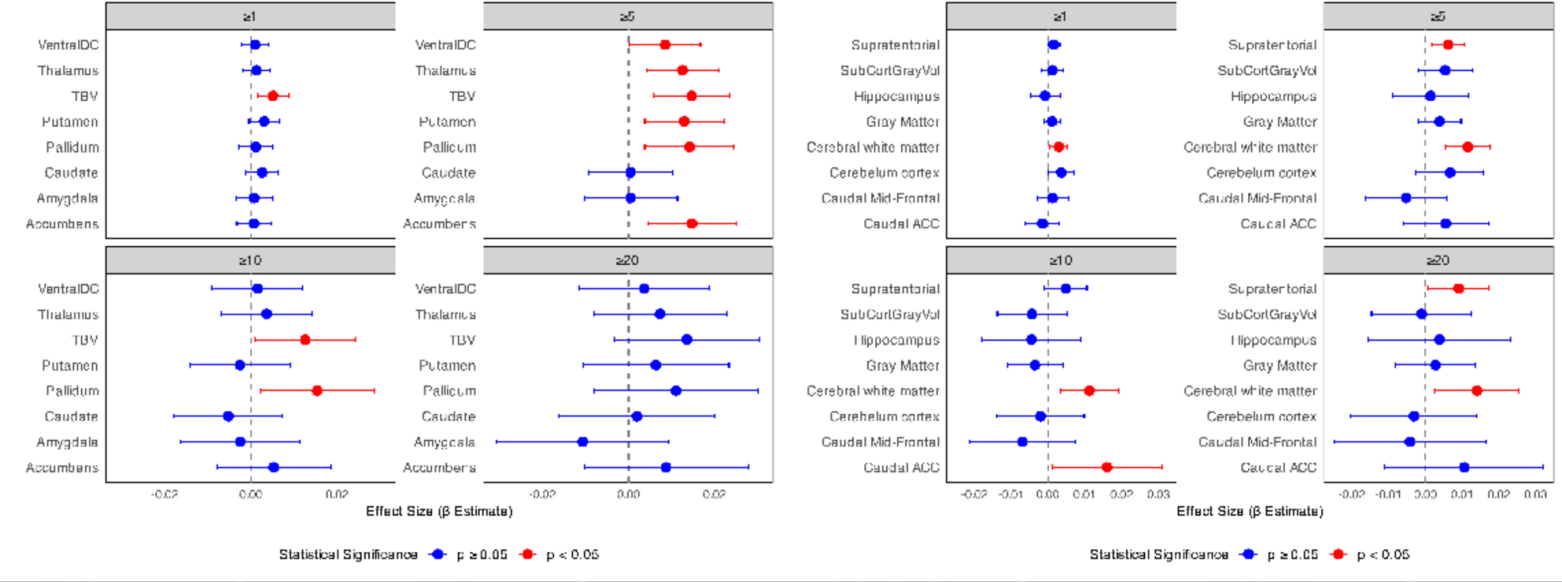
Polygenic STR expansion burden and brain phenotypes in the UK Biobank. Forest plot of standardized β with 95% confidence intervals for the association between polygenic STR expansion burden and brain phenotypes in the UK Biobank replication cohort. Each point corresponds to the β estimate for a given brain region at a specific STR threshold, with horizontal bars indicating the 95% CI. Associations that remained after correction for multiple testing using the Li & Ji are shown in red whereas non-significant associations are shown in blue. The vertical dashed line represents the null effect (β = 0). Statistically significant replicated associations were observed for TBV and thalamic volume for STR expansion burden defined as ≥ 5 repeat units above the reference. Abbreviations: TBV, total brain volume; ventral DC, ventral diencephalon; caudal ACC, caudal anterior cingulate cortex

In both cohorts, as the expansion threshold increased, the number of STR expansions per individual decreased, highlighting the relative rarity of large expansions in the population (Additional file 1: Fig. S5). In the discovery cohort, we identified significant associations between polygenic STR burden and increased brain volumes across multiple global and regional measures. After correcting for multiple testing using the Li and Ji method [63], we found that individuals with a higher polygenic STR burden (≥ 5 repeat units longer than the reference sequence) exhibited significantly larger volumes in key brain regions, including the thalamus (β = 0.05, 95% CI [0.02 to 0.07]), total brain ; β = 0.02, 95% CI [0.007 to 0.03]), and total gray matter (β = 0.03, 95% CI [0.01 to 0.04]) (Fig. 4). These associations remained statistically significant also at additional thresholds (≥ 10 and ≥ 20 repeats) (Fig. 5). Further associations were observed for supratentorial volume, subcortical gray matter volume, and cerebellar cortex volume at the ≥ 10 and ≥ 20 repeat thresholds. At the regional level, polygenic STR burden (≥ 10 repeats) was significantly associated with larger cortical thickness in the left rostral anterior cingulate cortex and left medial orbitofrontal cortex (Additional file 1: Fig. S8). These effects were consistent and remained statistically significant after multiple-testing correction using the Li & Ji approach. In permutation-based sensitivity analyses, the empirical family-wise error rate threshold in the discovery cohort was α = 5.51 × 10⁻⁴. Ten of the fifteen associations identified using the Li & Ji correction remained significant under this more stringent threshold, including the principal thalamic and total brain volume association (≥ 5 repeats), while the other association remained nominally significant.

We again tested whether the associations between polygenic STR expansion burden and brain phenotypes reported above were directionally consistent in the UKB cohort. Consistent with our discovery results, we found that individuals with a higher polygenic STR expansion burden (≥ 5 repeat units longer than the reference sequence) exhibited significantly larger brain volumes. Specifically, we observed directionally consistent positive associations for total brain volume; β = 0.005, 95% CI [0.002 to 0.008], thalamic volume (β = 0.001, 95% CI [0.0004 to 0.002]) and supratentorial volume (β = 0.006, 95% CI [0.0001 to 0.001]) (Fig. 5). For the remaining imaging-derived phenotypes, effect directions were broadly concordant with those observed in the discovery cohort, with the majority showing nominal statistical support (*p* < 0.05) (Additional file 1: Fig. S9).

Using the threshold-based approach, we observed consistent trends of increasing STR burden with moderate expansion magnitude across several brain phenotypes. However, given the limitations of threshold-based definitions, we further evaluated a continuous, weighted STR burden as a complementary analysis. Specifically, we restricted the analysis to STR loci significantly associated with brain morphology phenotypes (*p* < 0.05) and constructed a weighted burden score for each individual. This score was calculated as the sum of STR dosages weighted by their corresponding standardized effect size estimates derived from association analyses.

The continuous STR burden score showed robust positive associations with multiple subcortical and global brain volume measures in the Rhineland Study. Significant associations were observed for the thalamus, putamen, ventral diencephalon, subcortical gray matter, supratentorial volume, amygdala, and total brain volume (all FDR-p-value < 0.05). These associations were directionally consistent and statistically significant in the UKB. In addition, associations observed for cortical regional measures in the Rhineland Study showed consistent directions of effect in the UKB, further supporting the robustness and reproducibility of these findings. We note that loci included in the burden score were selected based on statistical significance, which may introduce upward bias in effect size estimates (winner’s curse). Accordingly, these analyses are presented as complementary to the primary framework and are reported in the Additional file 1: Figs. S10 and S11.

### Age-dependent effects of polygenic STR burden on brain volume

**Fig. 6:**
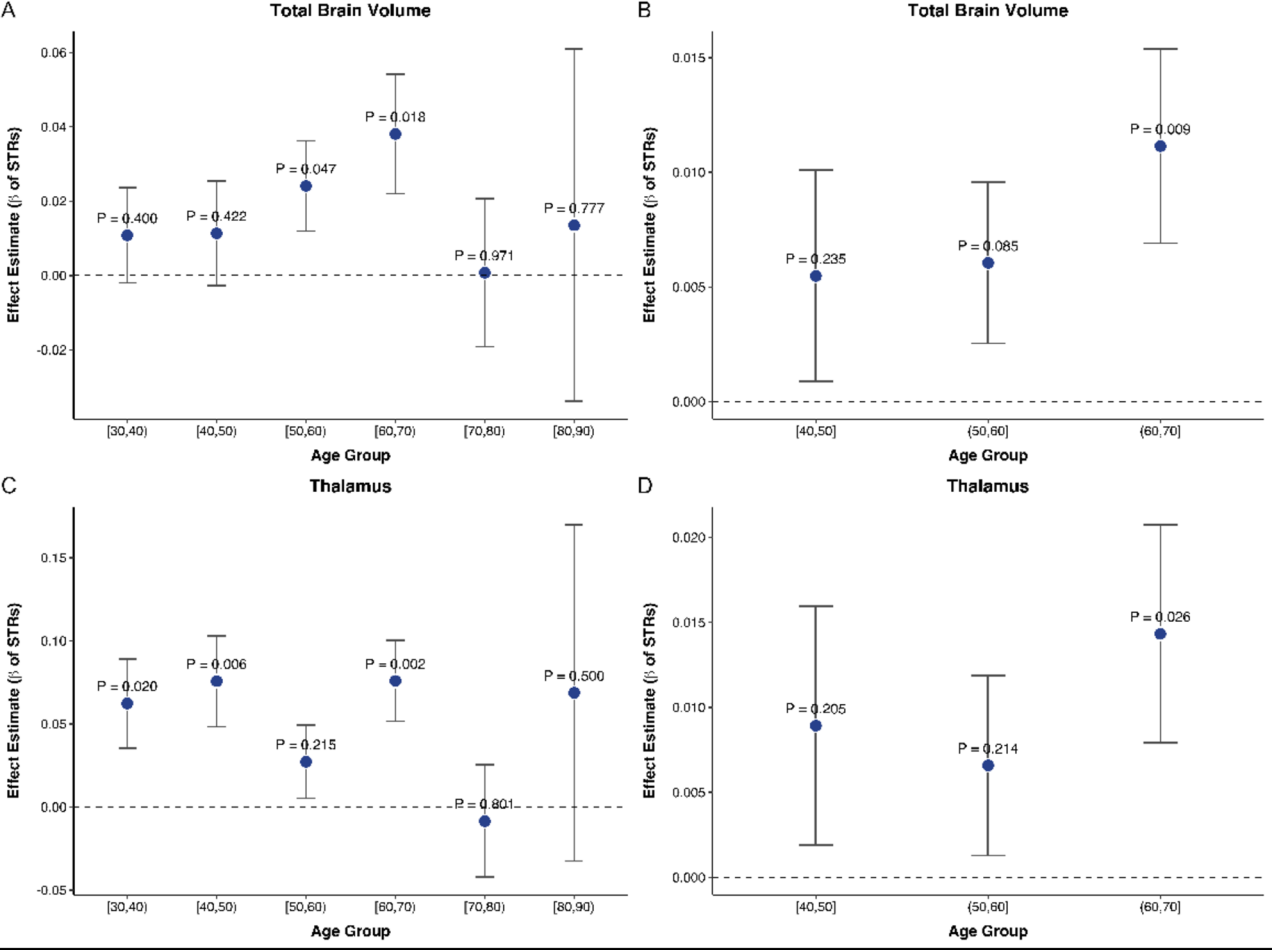
Age-stratified effects of STR expansion burden on total brain volume (TBV; panels **A**, **B**) and thalamic volume (panels **C**, **D**). Results shown for Rhineland Study (**A**, **C**) and UK Biobank (**B**, **D**). Participants grouped into 10-year age bins (based on age ranges of the respective cohorts)

Given that STRs are prone to genomic instability, which can lead to repeat expansions with increasing age, we examined the age-dependent effects of polygenic STR burden on brain volume, focusing on the associations that replicated. Participants were grouped into 10-year age bins within each cohort based on age at imaging acquisition, and effect sizes for the ≥ 5 repeat expansion threshold were evaluated across these age groups. Interestingly, the relationship between polygenic STR burden and both total brain volume and thalamic volume followed a non-linear trend, with the strongest effects observed between ages 60 and 70 (Fig. 6). In the Rhineland Study, the effect size declined after age 70, although this change was not statistically significant, while in the UKB cohort, this could not be evaluated due to the upper age limit of participants.

### Pathway enrichment of genes with moderately expanded STR

To better understand the biological mechanisms mediating the relation between STRs and brain structure, we carried out gene ontology (GO) and pathway enrichment analyses at different levels of repeat expansion thresholds. Prior to this, we conducted a genome-wide annotation of approximately 1.2 million STRs identified in the GRCh38 human reference genome [41]. We then performed a proximity search against annotated human genes from the UCSC Genome Browser (GRCh38 reference genome) to identify the nearest gene for each STR locus. We considered STRs located within a maximum distance of 250 kb from a gene’s transcription start site (TSS) for further analysis. We next ranked the genes in our customized STR panel at predefined expansion thresholds based on carrier frequency within each cohort. At each threshold (≥ 1, ≥ 5, ≥10, or ≥ 20 repeat units longer than the reference), we identified the most frequent genes shared between cohorts and used these for gene set enrichment analysis.

**Fig. 7:**
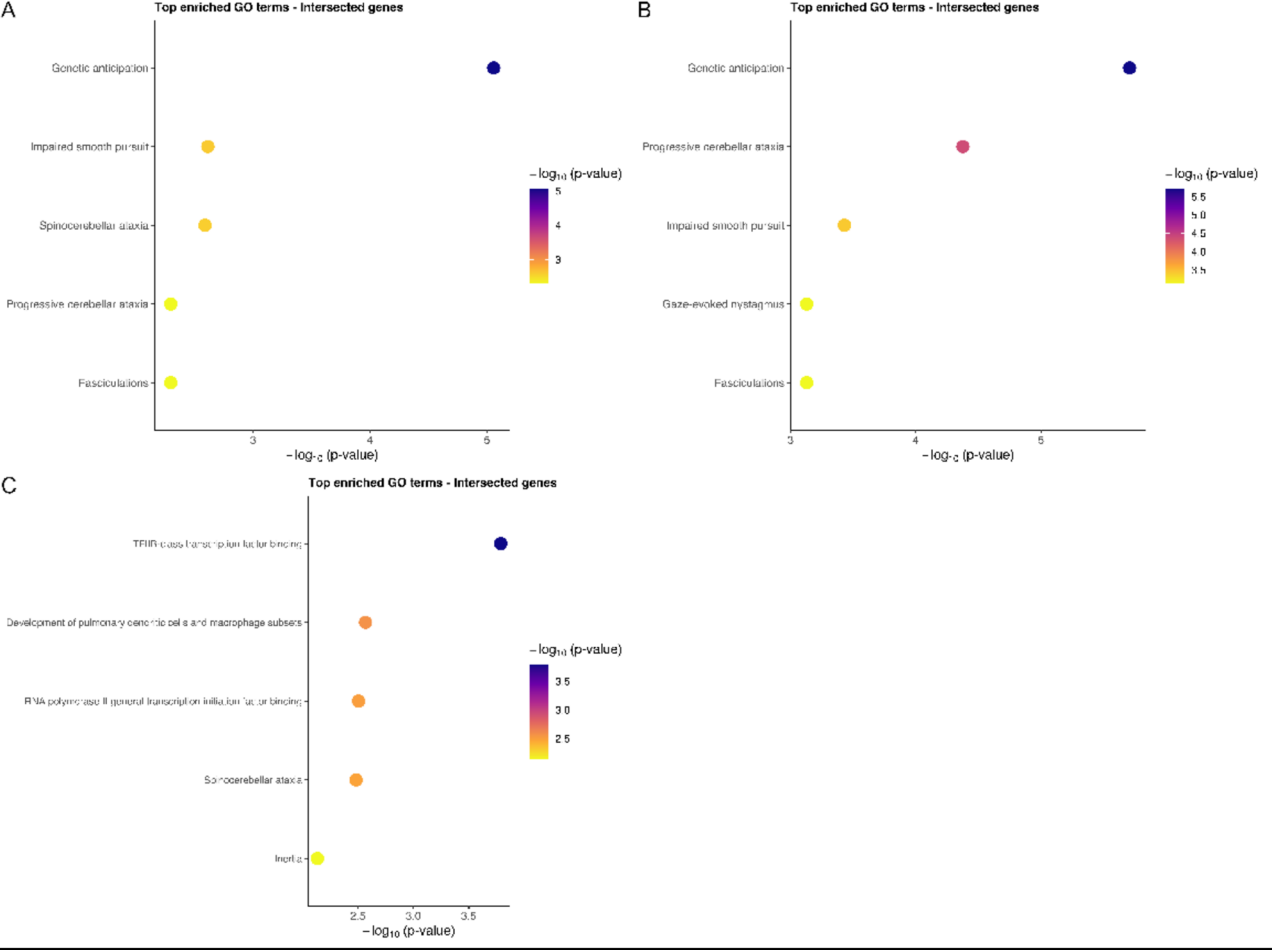
Pathway enrichment results across STR expansion thresholds. Figure shows pathway enrichment results for genes linked to STR expansions at three thresholds (≥ 5, ≥ 10, ≥20 repeat units). **A**,** B** and **C**: Enrichment results at ≥ 5, ≥10 and 20-unit thresholds, respectively. The x-axis indicates enrichment significance (− log₁₀ *p*-value), while the y-axis indicates the pathway/term, with bubble size representing the relative number of genes in each pathway

In our enrichment analysis, we identified sets of enrichment terms that were consistent across the moderate expansion thresholds. At the ≥ 5 repeat units’ threshold, the common top enriched terms were genetic anticipation, spinocerebellar ataxia, progressive cerebellar ataxia, impaired smooth pursuit, and fasciculations, which suggest convergence on neurological features (Fig. 7). At ≥ 10 units threshold, the enrichment terms genetic anticipation and progressive cerebellar ataxia remained and expanded to include gaze-evoked nystagmus and fasciculations. At the highest threshold of ≥ 20 units, however, the overlap shifted toward molecular and cellular processes. The enrichment terms included TFIIB-class transcription factors, RNA polymerase II transcription initiation, and development of pulmonary dendritic cells and macrophages, alongside neurological terms such as spinocerebellar ataxia (Fig. 7).

To further explore potential functional mechanisms, we assessed overlap between identified STR loci and previously reported expression-associated STRs (eSTRs) from GTEx brain tissues [7]. No direct overlap or proximity (± 10 kb) was observed, suggesting that the identified loci are not captured by current eSTR resources.

## Discussion

We found an intriguing pattern of associations of both specific STR loci as well as overall STR burden across the genome with brain structure. Moreover, whereas some STR loci demonstrated global effects on brain volume, others exhibited region-specific effects, indicating a key role for STRs in the modulation of brain structure. Specifically, we observed that individuals with a longer AC motif in an intronic region of the *PRR14L* gene had significantly larger thalamic volume, while repeat number variations of an AATG motif in *NADK* was significantly associated with reduced subcortical gray matter volume and thalamic volume. Interestingly, we also found that a higher repeat expansion burden, even just 5 units longer than the human reference, was consistently associated with larger volumes across multiple brain regions.

We found that STR variations in the *PRR14L* gene were associated with larger thalamic volume. Even though the function of *PRR14L* in neuronal tissues is yet to be defined, prior studies demonstrated that it is involved in cell division and fate specification [65]. This raises the possibility that STR variations could influence thalamic development through altered gene regulation or neuronal differentiation. This finding aligns with emerging literature demonstrating the impact of STRs on brain structure and risk for neurological disease [16, 66]. Given the importance of thalamic volume in sensory processing, cognition, and neuropsychiatric health [67], this warrants further investigation into the molecular mechanisms through which *PRR14L* STRs might affect neural development. Moreover, we found that STR polymorphisms in the *NADK* gene were associated with reduced subcortical gray matter and thalamic volumes. In the brain and other tissues, *NADK* is the sole enzyme responsible for phosphorylating NAD + to produce NADP+. This enzymatic step is critical for maintaining cellular redox homeostasis and supports numerous anabolic and metabolic processes that rely on NADPH-dependent pathways [68]. This finding suggests that there might be a genetic link between the central NAD+ metabolic pathway and structural brain changes. This link might be particularly relevant as dysregulation of NAD + and its derivatives has been implicated in aging and neurodegenerative disorders [69]. Moreover, the lack of overlap with known eSTRs suggests that the STRs identified here may not exert detectable effects on steady-state gene expression in the tissues and conditions represented in current GTEx datasets. Alternatively, their functional effects may be context-specific (e.g., dependent on cell type or developmental stage) or mediated through mechanisms not captured by bulk tissue expression analyses. Additionally, we assessed whether the two identified STR loci overlapped with annotated human accelerated regions (HARs) using the UCSC Genome Browser hg38 “Unusual Conservation” track. However, neither of the identified loci overlapped with known HAR elements, suggesting that the observed associations are unlikely to be mediated through currently annotated HAR regions.

In the last two decades, STRs have increasingly been recognized for their substantial contribution to the genetic basis of many traits across diverse species. Multiple studies have demonstrated that non-pathogenic STR polymorphisms modulate functional traits [70, 71]. Among the most well-studied STR loci are those located in the period gene of Drosophila melanogaster (thought to participate in circadian rhythmicity), the *Avpr1a* gene of voles (reported to be involved in social recognition memory, aggression and sociosexual behaviours in vertebrates), and the *SLC6A4* gene (implicated in the multiple aspects of primate behaviour) [72–74]. Moreover, variation in STR lengths within key developmental genes such as *Runx-2* and *Alx-4* have been associated with striking differences in skull shape and limb morphology among dog breeds [75, 76]. Together, these examples underscore STR variation as a potent mechanism for rapid and flexible phenotypic evolution across species.

Despite advances in animal models, research on the impact of STRs on human brain morphology is scarce, particularly regarding the polygenic burden of STRs across genes with unknown pathogenic thresholds. Our study provides novel evidence that even modest expansions of STRs, within the non-pathogenic range, are associated with increased brain volume (both global and regional) in healthy individuals. Remarkably, we found that increases of even just five STR units in the targeted genomic regions were associated with structural benefits. This association suggests that STRs at sub-pathogenic repeat lengths may promote advantageous developmental changes, potentially by modulating gene expression or protein function during early neurodevelopment [7]. Indeed, our findings demonstrate that STRs, once referred to as “junk DNA”, are important genetic determinants of brain structure, and thereby may also contribute to human neurodiversity.

An additional intriguing aspect of our results is the clear age-related pattern of STR effects on the brain. In both cohorts, the association between polygenic STR burden (for moderate expansions) and brain volume increased steadily with age, with peak effect size occurring between ages 60 and 70 years. The age-dependent findings suggest that STRs may confer beneficial effects on brain structure during mid- to late adulthood, but that their influence could diminish or even become detrimental at older age. This age-dependent shift aligns with the concept of antagonistic pleiotropy, where genetic variants confer advantages early in life but may lead to adverse outcomes later on [15, 77]. For STRs, this could mean that moderate expansions support healthy brain development and function. Over time, however, the accumulation of additional repeat units with age, potentially driven by ongoing mutations or somatic instability, may ultimately shift the balance. This concept is supported by findings from the Kids-HD study, which followed children and young adults carrying pathogenic CAG expansions in the *HTT* gene [13]. Despite their increased risk for HD later in life, these individuals demonstrated significantly better cognitive, behavioral, and motor performance during youth compared to non-carriers. These results support the notion that pathogenic repeat expansions in the *HTT* gene may confer neurodevelopmental advantages early in life, even though these same expansions ultimately lead to deleterious effects in adulthood. It is therefore similarly conceivable that extreme repeat expansions in the genes examined here might also produce detrimental effects with advanced age, albeit at a slower pace compared to alleles in the pathogenic range.

Our enrichment analysis revealed that moderate repeat expansions, common among many participants, were primarily associated with neurological traits as well as several fundamental molecular and cellular processes. This suggests that the spectrum of biological effects of these STRs may depend on expansion size. Notably, we observed enrichment of “genetic anticipation” gene sets among modestly expanded STR loci. This supports the idea that even repeat lengths below the known pathogenic thresholds can engage biological pathways typically linked to repeat expansion disorders [78]. Intriguingly, individuals with moderately expanded repeats (≥ 5 units longer than the reference) exhibited increased brain volumes in mid- to late adulthood. Given that this effect diminishes with age, these alleles may exert subtle, age-dependent influences on neurodevelopment and aging, mirroring mechanisms involved in genetic anticipation but without reaching pathological levels. While we focused on STRs within 250 kb of gene TSS to prioritize potentially regulatory loci, future studies integrating three-dimensional genome organization data may identify additional STRs exerting long-range or trans-regulatory effects beyond linear genomic proximity. Additionally, integrating single-cell eQTL resources or epigenomic annotations may provide further insight into the functional role of these loci.

An important consideration is whether the observed STR associations reflect causal effects or arise due to linkage disequilibrium (LD) with nearby variants. To address this, we examined common SNPs within ± 250 kb of the lead STR loci, but did not identify significant SNP associations in these regions. While this supports the possibility that the STR signals are not simply tagging nearby common SNP effects, it does not establish causality. LD with untyped, rare, or poorly imputed variants, as well as other forms of structural variation, cannot be excluded. Accordingly, our findings should be interpreted as evidence of association, and further work integrating fine-mapping, sequencing, and functional assays will be required to clarify the underlying causal mechanisms.

Among the moderately expanded STRs consistently associated with brain structural measures was one locus within the runt-related transcription factor 2 (*RUNX2*) gene. *RUNX2* encodes a master transcription factor that acts like a “switch” and regulates essential processes such as ossification, cranial suture closure, and bone morphogenesis [79]. Recent work in dogs revealed that STR variations in *RUNX2* correlate with distinct differences in limb and skull morphology [80]. Interestingly, *RUNX2* is expressed in key regions of the human brain such as the cerebellum, hippocampus and neocortex (https://www.proteinatlas.org/ENSG00000124813-RUNX2/brain; accessed on July 28, 2025), suggesting that this gene may be implicated in neurogenesis and neuronal function. Our findings further imply a potential role for *RUNX2* in neuronal function via its STR domain, demonstrating that non-pathogenic STR variation is biologically relevant.

Some association signals observed in the Rhineland Study cohort could not be replicated in the UKB. A key factor likely contributing to this discrepancy is the technical difference in sequencing read length between the two datasets. In the Rhineland Study we used a targeted sequencing approach with relatively long paired-end reads (2 × 250 bp) with a high coverage (~ 180) per locus to obtain more accurate estimates of STR sizes, whereas in the UKB cohort WGS with shorter paired-end reads (2 × 150 bp) and a lower coverage (~ 30) was used. The relatively shorter read length and lower coverage per locus in the UKB cohort is likely to have influenced the accuracy of detecting longer repeat expansions, which could partly explain the limited directional consistency for certain associations. In addition, other differences between the two cohorts in demographic composition, especially age and ancestry, may also have contributed to some of the discrepancies. For example, the Rhineland Study sample had a wider age range distribution than the UKB sample, which may have led to underestimation of age-dependent STR effects in the latter. Additionally, the present study did not include the X chromosome, as analysis of sex-linked STR variation requires specialised approaches to account for ploidy, X-inactivation, and sex-specific allele dosage. Future sex chromosome-aware analyses may provide additional insight into the role of X-linked STRs in brain-related traits. Other limitations of our study include lack of longitudinal and cross-ancestry data, which will be subject of future research. Furthermore, emerging artificial intelligence-driven neuroimaging approaches, including those still in development, may improve the resolution and statistical power of future STR association analyses.

## Conclusions

In summary, our work demonstrates that STR polymorphisms can act as important genetic modifiers of brain structure in the general population. Moreover, our findings support the antagonistic pleiotropy model for STRs, indicating that highly polymorphic STR loci may have evolved due to their positive contributions to neuroanatomical plasticity and cognitive function. Future studies exploring the mechanistic basis of these effects are warranted. This especially includes investigating how subtle STR expansions influence gene expression and neurodevelopment. Taken together our work lays the foundation for integrating STR analysis into studies of age-associated brain health and neurodegenerative diseases.

## Supplementary Information

Additional file 1. Supplementary figures and tables. This file contains all supplementary figures and tables supporting the main findings, including extended analyses and additional results that are not included in the main manuscript.

Additional file 2. Genomic coordinates of unannotated significant loci. This file provides an Excel dataset containing the genomic coordinates of significant loci without gene annotations. These loci were excluded from figure labeling for visual clarity.

Additional file 3. Selected STR loci and annotations. This Excel file contains all selected STR loci, including genomic coordinates, associated gene annotations, and selection category.

## Data Availability

Due to data protection regulations, the Rhineland Study data are not publicly available. However, qualified researchers may request access in accordance with the Rhineland Study’s Data Use and Access Policy. Requests for data access or additional information can be directed to rs-duac@dzne.de. All whole-genome sequencing as well as individual-level data from the UK Biobank Imaging Study used in this work are available through the UKB Resource (https://www.ukbiobank.ac.uk). Access to these data is subject to UK Biobank’s data access policies. Part of this research has been conducted using the UK Biobank Resource under Application Number 82056. All data are available in the main text or the supplementary materials.

## Abbreviations

BWA: Burrows–Wheeler Aligner
DBSCAN: Density-Based Spatial Clustering of Applications with Noise
eTIV: Estimated total intracranial volume
FDR: False discovery rate
FASTQ: FAST Quality format (text-based sequencing file format)
GO: Gene Ontology
GWAS: Genome-wide association study
ICH-GCP: International Council for Harmonisation – Good Clinical Practice / Declaration of Helsinki
KEGG: Kyoto Encyclopedia of Genes and Genomes
NADK: NAD kinase
PCs: Principal components
PDAG: Polyglutamine disease–associated genes
PRR14: Proline-rich protein 14
REDs: Repeat expansion disorders
RINT: Rank-based inverse normal transformation
SNPs: Single nucleotide polymorphisms
STR: Short tandem repeat
TBV: Total brain volume
UKB: UK Biobank
WGS: Whole-genome sequencing
